# A novel experimental design for the measurement of metacarpal bone loading and deformation and fingertip force

**DOI:** 10.7717/peerj.5480

**Published:** 2018-09-11

**Authors:** Szu-Ching Lu, Evie E. Vereecke, Alexander Synek, Dieter H. Pahr, Tracy L. Kivell

**Affiliations:** 1 Animal Postcranial Evolution Lab, Skeletal Biology Research Centre, School of Anthropology and Conservation, University of Kent, Canterbury, UK; 2 Department of Development and Regeneration, University of Leuven, Kortrijk, Belgium; 3 Institute of Lightweight Design and Structural Biomechanics, Vienna University of Technology, Vienna, Austria; 4 Department of Anatomy and Biomechanics, Karl Landsteiner Private University of Health Sciences, Krems an der Donau, Austria; 5 Department of Human Evolution, Max Planck Institute for Evolutionary Anthropology, Leipzig, Germany

**Keywords:** Hand, Biomechanics, Force, Strain

## Abstract

**Background:**

Musculoskeletal and finite element modelling are often used to predict joint loading and bone strength within the human hand, but there is a lack of in vitro evidence of the force and strain experienced by hand bones.

**Methods:**

This study presents a novel experimental setup that allows the positioning of a cadaveric digit in a variety of postures with the measurement of force and strain experienced by the third metacarpal. The setup allows for the measurement of fingertip force as well. We tested this experimental setup using three cadaveric human third digits in which the flexor tendons were loaded in two tendon pathways: (1) parallel to the metacarpal bone shaft, with bowstringing; (2) a semi-physiological condition in which the tendons were positioned closer to the bone shaft.

**Results:**

There is substantial variation in metacarpal net force, metacarpal strain and fingertip force between the two tendon pathways. The net force acting on the metacarpal bone is oriented palmarly in the parallel tendon condition, causing tension along the dorsum of the metacarpal shaft, while the force increases and is oriented dorsally in the semi-physiological condition, causing compression of the dorsal metacarpal shaft. Fingertip force is also greater in the semi-physiological condition, implying a more efficient grip function. Inter-individual variation is observed in the radioulnar orientation of the force experienced by the metacarpal bone, the fingertip force, and the strain patterns on the metacarpal shaft.

**Conclusion:**

This study demonstrates a new method for measuring force and strain experienced by the metacarpal, and fingertip force in cadaveric digits that can, in turn, inform computation models. Inter-individual variation in loads experienced by the third digit suggest that there are differences in joint contact and/or internal bone structure across individuals that are important to consider in clinical and evolutionary contexts.

## Introduction

Different manipulative and locomotor behaviours lead to varied biomechanical loading of the hand and affect the external morphology (e.g. entheses) and internal structure (e.g. cortical thickness, trabecular architecture) of bones. The ability of bone tissue to remodel in response to the magnitude and direction of load is known traditionally as Wolff’s law (Wolff, 1892) or, now more accurately, as bone functional adaptation (Cowin et al., 1985; Rubin & Lanyon, 1985; Ruff, Holt & Trinkaus, 2006). From this perspective, the morphology of the hand bones should reflect, at least to some degree, how an individual used their hands during life. Since bony (and dental) morphology is often the only part of an organism to preserve in the fossil record, the functional morphology of hand bones has played a central role in the understanding of major locomotor and manipulative transitions in the evolution of humans (Alba, Moyà-Solà & Köhler, 2003; Kivell, 2015; Marzke & Marzke, 2000; Napier, 1962; Tocheri et al., 2008) and other primates (Almécija et al., 2007; Almécija, Smaers & Jungers, 2015; Begun & Kivell, 2011; Bloch & Boyer, 2002).

With recent advances in 3D imaging techniques, the internal bone structure of human, nonhuman primate, and fossil human (hominin) hand bones has been investigated to help reconstruct hand function and behaviour in extinct taxa. Variation in cortical and trabecular bone structure has been linked to the differences in hand loading during locomotor behaviours in living apes vs. manipulation in humans (Lazenby et al., 2011; Marchi, 2005; Sarringhaus et al., 2005; Stephens et al., 2016; Tsegai et al., 2013, 2017; Zeininger, Richmond & Hartman, 2011). It has also been used to infer hand function in the past, including the evolution of tool-use behaviours and precision grip abilities in fossil hominins (Skinner et al., 2015; Stephens et al., 2018). However, these functional interpretations are almost always based on loose estimates of joint posture at a predicted, but often unknown, point of peak loading during different manipulative (Key, 2016; Rolian, Lieberman & Zermeno, 2011; Williams, Gordon & Richmond, 2012) and, particularly, locomotor behaviours (Richmond, 2007; Wunderlich & Jungers, 2009). Our understanding of human hand evolution requires more direct data on the loads experienced by the hand and the strain experienced by the bones, both in humans and nonhuman primates.

Understanding the loads experienced by the hands is also important within a clinical context, including research on joint disease (Goislard De Monsabert et al., 2014; Moulton et al., 2001; Qiu & Kamper, 2014), trauma (Prevel et al., 1995; Schuind et al., 1992), ergonomics (Aldien et al., 2005; Hall, 1997) and joint replacement (An et al., 1985; Weightman & Amis, 1982). There are numerous ethical and logistical challenges to directly measuring force acting on hand bones in vivo and thus previous studies have used a variety of methods to measure load in cadaveric specimens. For example, Moulton et al. (2001) embedded pressure-sensitive film within the trapeziometacarpal joint of human cadaveric thumbs to examine the joint loading, and Barker et al. (2005) measured bone strain in a human second metacarpal. However, both of these analyses required substantial disruption of the surrounding anatomical structures (Moulton et al., 2001) or the complete extraction of the bone from the hand (Barker et al., 2005). As far as we are aware, only one study has measured bone strain within cadaveric hands, in which the authors simulated striking with a fist (Horns, Jung & Carrier, 2015).

Because both in vivo and in vitro studies of hand bone loading present several technical challenges, musculoskeletal models are typically used to predict force during isometric hand functions, such as pinch and power grip tasks (An et al., 1985; Goislard De Monsabert et al., 2014; Qiu & Kamper, 2014; Weightman & Amis, 1982). The static equilibrium method is broadly applied in these analytical models, such that the muscle and joint force distribution is determined indirectly to balance an external force applied at the fingertip (An et al., 1985; Goislard De Monsabert et al., 2014; Weightman & Amis, 1982), or fingertip force is predicted by simulated muscle force (Qiu & Kamper, 2014; Valero-Cuevas, Towles & Hentz, 2000). However, the ratio of the predicted joint force to the fingertip force varies substantially across studies (An et al., 1985; Goislard De Monsabert et al., 2014; Weightman & Amis, 1982), highlighting the importance of more direct measures of bone loading. In addition, finite element (FE) modelling has been used to estimate the mechanical response of the hand bones under specific loading conditions to better design prostheses (Barker et al., 2005; Butz, Merrell & Nauman, 2012a, 2012b) or to understand the functional biomechanics of curved hand bones (Nguyen et al., 2014; Richmond, 2007). Knowledge of the actual forces occuring in the hand during various functional tasks is critical to the robustness of musculoskeletal and FE modelling, and yet these forces are largely unknown and models were rarely compared with in vivo or in vitro experimental data (An et al., 1985; Barker et al., 2005). Musculoskeletal and FE modelling are also becoming increasingly popular methodologies in palaeoanthropology to reconstruct the behaviour in the past (Domalain, Bertin & Daver, 2017; Nguyen et al., 2014; Rayfield, 2007). However, models are only useful if they can be shown to accurately reflect the biological conditions in extant taxa (Strait et al., 2005).

The purpose of this study is to help to fill this gap of information on in vitro hand bone loading. Here, we present a novel experimental setup that allows simultaneous measurement of in vitro loading and deformation of human and nonhuman primate hand bones during functional postures. The experimental setup also allows the measurement of fingertip force. This experimental design builds upon previous in vitro studies that typically only measure fingertip force in cadaveric specimens (Lee et al., 2008; Qiu & Kamper, 2014; Valero-Cuevas, Towles & Hentz, 2000). We test this experimental setup on three human cadaveric third digits and provide the net force and strain experienced by the metacarpal, as well as fingertip force; data that are critical for the informing musculoskeletal and FE hand models.

## Materials and Methods

### Experimental system

**Figure 1:**
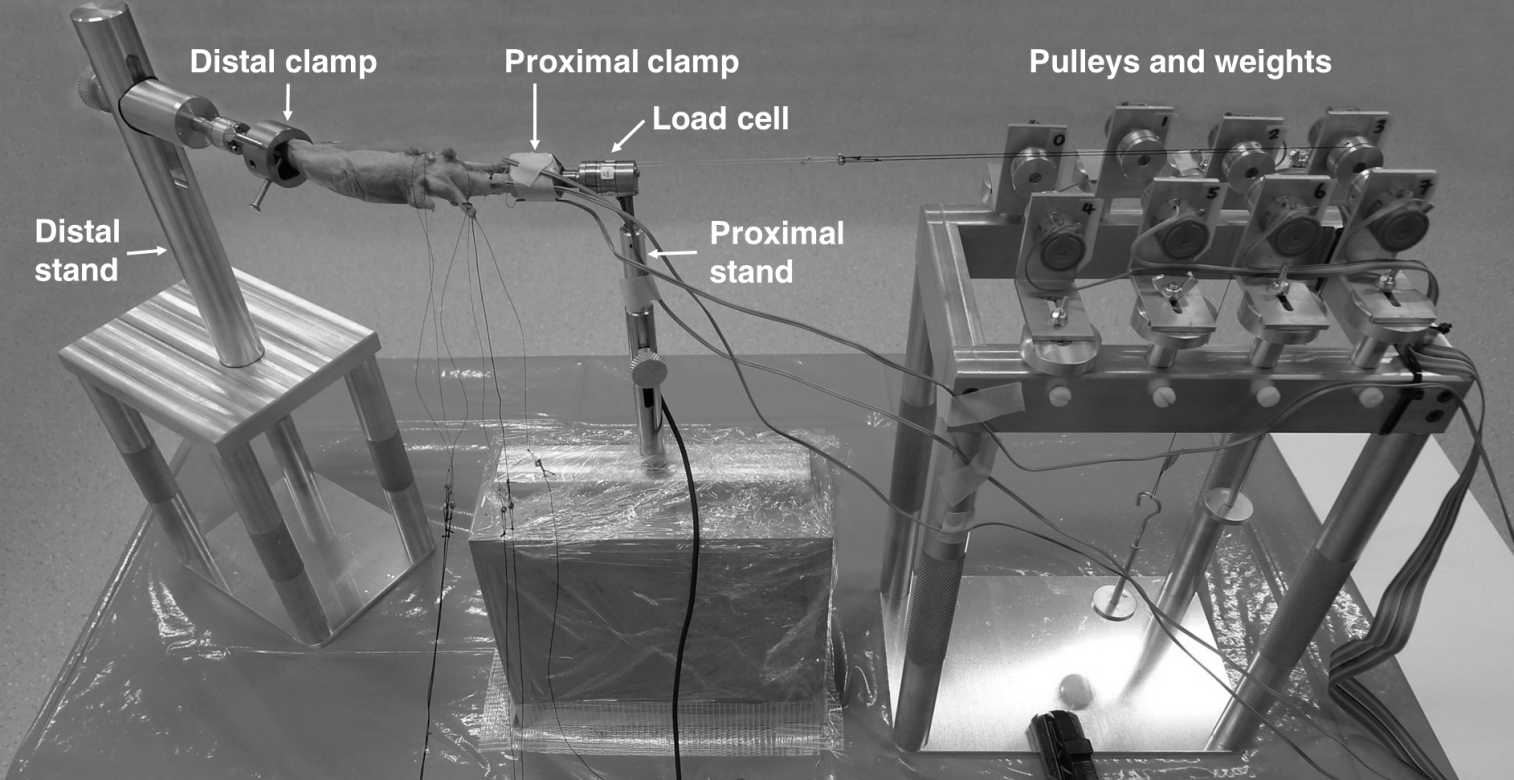
Experimental setup. A customized experimental setup was designed to fix the fingertip and the base of metacarpal bone using the distal and proximal clamps, respectively. A six-axis load cell was fixed onto the proximal clamp and the proximal stand to measure the force experienced by the metacarpal bone, and a set of pulleys and weights were used to load the tendons to simulate the muscle contraction during hand function. Photo credit: Dr. Szu-Ching Lu.

**Figure 2:**
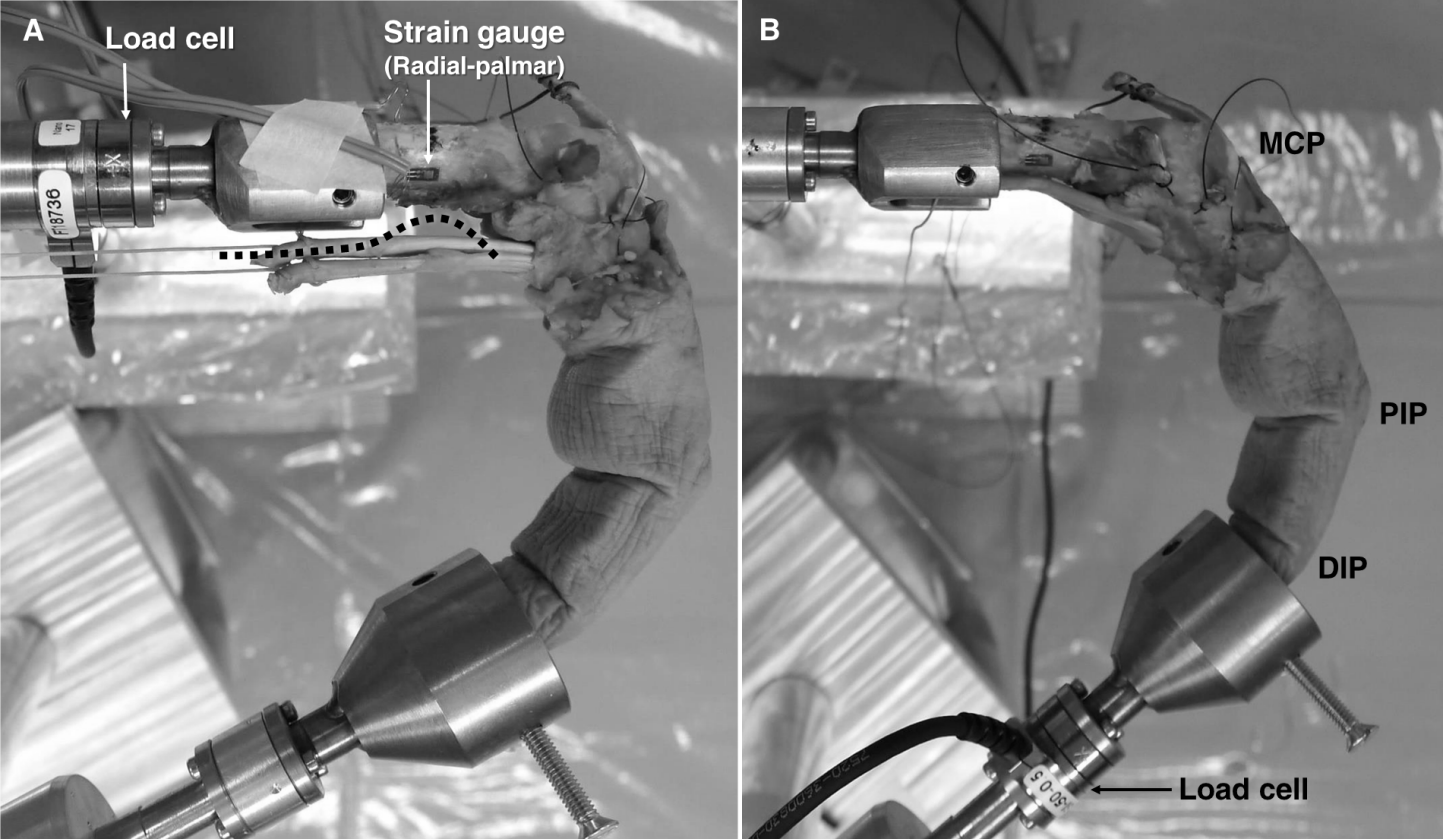
Metacarpal loading and deformation measurement and fingertip force evaluation. (A) The load cell was attached to the proximal clamp to measure the force experienced by the metacarpal bone, and the strain gauges were attached to the metacarpal shaft to measure the bone deformation. The third digit was in a flexed posture with the flexor tendons loaded and guided parallel to the bone shaft, and then a low-friction metal bar was applied to place the tendons in a semi-physiological pathway (the dash line). (B) The load cell was fixed to the distal clamp for the fingertip force measurement. DIP, distal interphalangeal joint; PIP, proximal interphalangeal joint; MCP, metacarpophalangeal joint. Photo credit: Dr. Szu-Ching Lu.

Two clamps were designed to fixate a cadaveric digit at its fingertip and its metacarpal base. The concave cavities of the clamps were designed to hold the fingertip and the metacarpal base of the specimen (see the distal and proximal clamps in Fig. 1). Two threaded holes with screws were fixed within each clamp to stabilize the specimen but without damaging the bone. Three pin holes in the outside base of each clamp were made for the attachment of either the height-adjustable metal stand or the force-measuring side of a load cell (Nano17-E; ATI Industrial Automation, Apex, NC, USA). The force-measuring side of the load cell could be fixed to the proximal clamp and the distal clamp for measuring the net force experienced by the metacarpal bone and the fingertip force, respectively (Figs. 1 and 2). The mounting side of the load cell could be fixed to the distal and proximal metal stands. The position of the distal metal stand was adjustable, allowing the digit to be moved in a variety of functional postures.

The experimental setup also included eight pulleys for loading tendons to simulate muscle contraction during hand function (Fig. 1). Each pulley could be adjusted to a different vertical and horizontal position, such that the tendon threads (see below) and weights did not interfere with each other.

Only one load cell was used in this study and therefore the net force experienced by the metacarpal bone and the fingertip force had to be measured separately. To measure the metacarpal force, the load cell was attached to the proximal clamp and the proximal metal stand. During the metacarpal force measurement, the bone deformation was quantified at the same time using strain gauges (FLA-1-11-1L; Tokyo Sokki Kenkyujo Co., Ltd., Tokyo, Japan). A compact data acquisition system (NI cDAQ-9174; National Instruments, Austin, TX, USA) and a customized program (LabVIEW; National Instruments, Austin, TX, USA) were designed to acquire the metacarpal force and strain signals simultaneously. To measure the fingertip force, the load cell was attached to the distal clamp and the distal metal stand. The same data acquisition system was used to acquire the fingertip force data.

### Specimens and preparation

**Figure 3:**
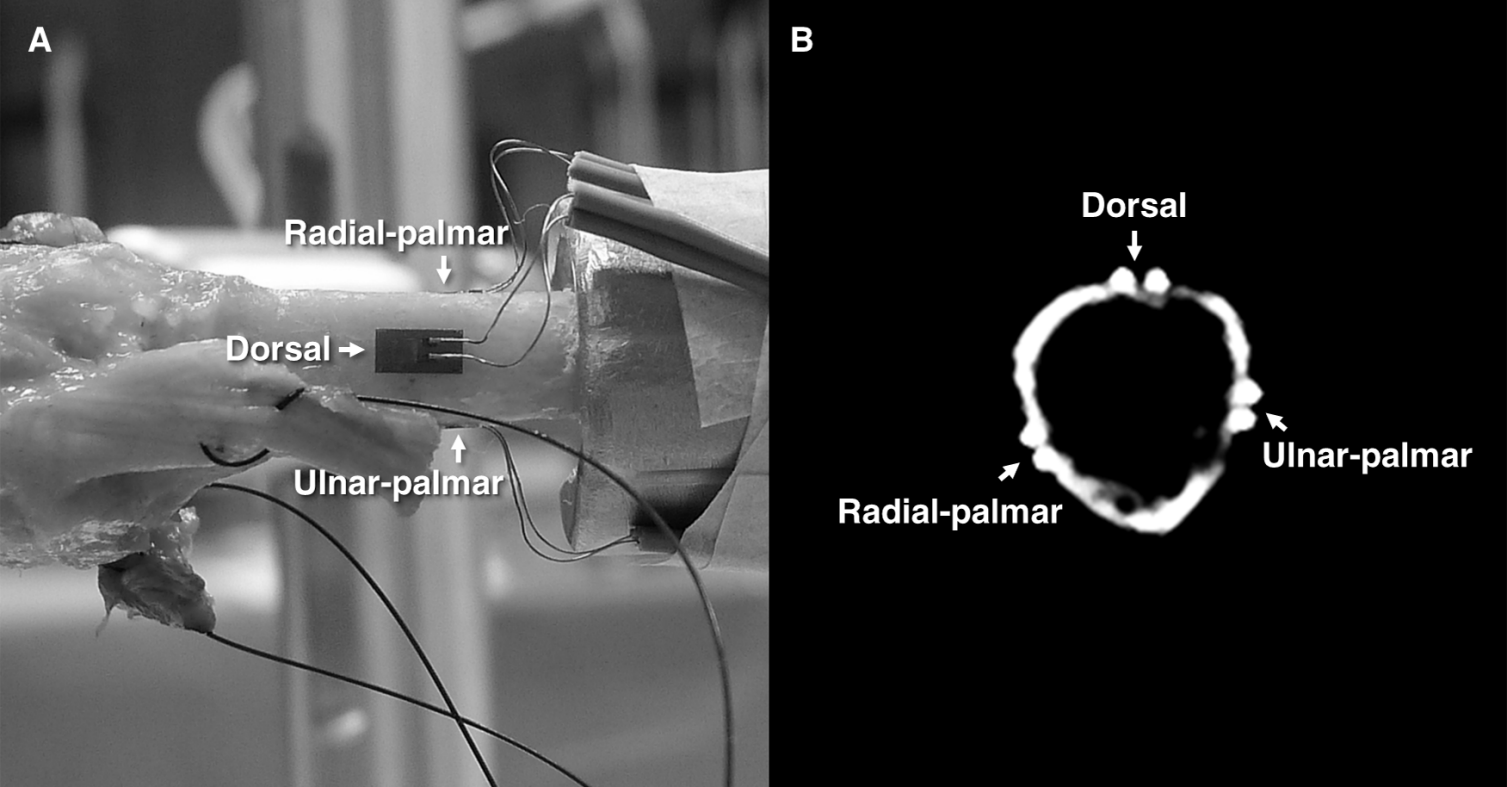
Stain gauge attachment. (A) Three strain gauges were applied to the radial-palmar, dorsal and ulnar-palmar sides of the metacarpal bone at its midshaft to quantify the bone deformation. (B) A computed tomography image shows the coronal cross-sectional view of the metacarpal bone with three strain gauges attached. Photo credit: Dr. Szu-Ching Lu.

Although the experimental setup can be used for each digit, we tested the setup on the third ray as this digit plays an important role in the daily activities of humans (Braido & Zhang, 2004; Key, 2016; Kuo et al., 2009; Marzke & Shackley, 1986; Williams, Gordon & Richmond, 2012) and nonhuman primates (Marzke & Wullstein, 1996; Neufuss et al., 2017; Samuel et al., 2018; Wunderlich & Jungers, 2009). Three fresh frozen human cadaveric left hands were obtained through the Human Body Donation Programme from the Medical Faculty of the University of Leuven, Belgium. The cadaveric hands were taken from one male (84-year-old) and two female donors (92- and 93-year-old). The third digit of each specimen was disarticulated from the rest of the hand at the carpometacarpal joint. The extrinsic flexor and extensor tendons were cut at approximately the point of the metacarpal midshaft. All the soft tissues from the metacarpophalangeal (MCP) joint to the fingertip were kept intact to preserve, as much as possible, the physiological condition of the finger. However, soft tissues surrounding the third metacarpal, including the intrinsic muscles (i.e. dorsal and palmar interossei) were carefully detached to expose the bony surface for the attachment of the strain gauges. The metacarpal bone surface was cleaned with acetone and three strain gauges were attached with a cyanoacrylate adhesive to the radial-palmar, dorsal and ulnar-palmar sides of the bone at its midshaft (Fig. 3). Nonelastic sutures were tied to the proximal ends of the flexor digitorum superficialis (FDS) and flexor digitorum profundus (FDP) tendons using the clove-hitch technique (Abraham et al., 2012).

### Experimental procedure

With the fingertip and the base of metacarpal bone fixed, each specimen was positioned with its distal interphalangeal (DIP) joint at 25° flexion, the proximal interphalangeal (PIP) joint at 60° flexion, and the MCP joint at 55° flexion as this joint configuration is at the approximate midpoint of the functional range of motion required to perform 90% of daily manipulative activities (Bain et al., 2015; Lee & Jung, 2015). In addition, the MCP joint was maintained at 0° radioulnar deviation. The joint angles were confirmed across experiments and individuals using goniometers. The load cell was fixed to the proximal clamp and the proximal stand to measure the net force experienced by the metacarpal bone (Fig. 2A). The FDS and FDP tendons were loaded as these muscles are responsible for the majority of finger grip force (An et al., 1985; Gurram, Rakheja & Gouw, 1995; Yang et al., 2009). In humans, the FDS and FDP muscles have similar physiological cross-sectional area (PCSA) values (Chao et al., 1989; Lieber et al., 1992; Ward et al., 2006). Each of the FDS and FDP tendons was loaded with the weight of 300 grams in this study, which was approximately 3% of the maximum muscle force derived with a specific tension of 22.5 N/cm^2^ (Powell et al., 1984) and a PCSA of 4.2 and 4.1 cm^2^ for the FDS and FDP, respectively (Chao et al., 1989). This specific load was chosen to avoid damaging the specimen and to allow modification of the tendon pathway (see below), but to also provide enough force for which strain of the metacarpal bone could be registered.

Unlike previous studies that only measured the fingertip force in cadaveric specimens (Lee et al., 2008; Qiu & Kamper, 2014; Valero-Cuevas, Towles & Hentz, 2000), we needed to attach strain gauges to the metacarpal bone to measure bone deformation, which required detachment of intrinsic muscles, disrupting the physiological tendon pathway. Therefore, during the experiment, the flexor tendons were guided along two different pathways: (1) parallel to the proximodistal axis of the metacarpal bone, which, given the lack of soft tissue constraints, allowed the tendons to bowstring when the finger was flexed and, (2) a semi-physiological tendon pathway in which the tendons were artificially positioned close to the bone, but without touching the metacarpal shaft, using a low-friction 14 mm diameter metal bar (Fig. 2A). We assume that these two tendon paths represent the two extreme conditions and that the range of results will encompass the physiological condition of metacarpal force and strain. Furthermore, although the parallel tendon pathway is a simplified condition that is not fully representative of the biological condition, it is particularly valuable for building informative musculoskeletal or FE models and obtaining replicable predictions. In contrast, the semi-physiological condition provides the best possible approximation of the normal physiological state within the constraints of experimental conditions.

Following the quantification of the force and strain experienced by the metacarpal, the strain gauge wires were removed and the load cell was fixed to the distal clamp and the distal stand for the direct measurement of fingertip force. The same posture, loading conditions and tendon pathways were applied as described above.

### Data acquisition and analysis

First, the force and strain experienced by the metacarpal bone were collected synchronously at 100 Hz, and then just fingertip force data was acquired at 100 Hz. The acquired force and strain signals were calibrated with baseline data collected when the flexor tendons were not loaded. Then, the signals were filtered using a sixth-order Butterworth low-pass filter with the cut-off frequency at 6 Hz. For the parallel condition, the average force and strain values within the middle 3 seconds of data collection were calculated. For the semi-physiological condition, a single frame of data was extracted since the tendon path was dynamically modified. All analyses were conducted within a customized MATLAB script (The MathWorks, Inc., Natick, MA, USA; see Supplemental Information). The force data are presented in the dorsal-palmar, proximal-distal, and radial-ulnar directions with respect to either the third metacarpal or distal phalanx.

## Results

The force and strain results varied substantially between the two tendon path conditions and, in some cases, across individuals. Below we present the results of net force and strain experienced by the metacarpal bone and fingertip force of the three test subjects in each tendon path condition.

### Net force experienced by the metacarpal bone

**Figure 4:**
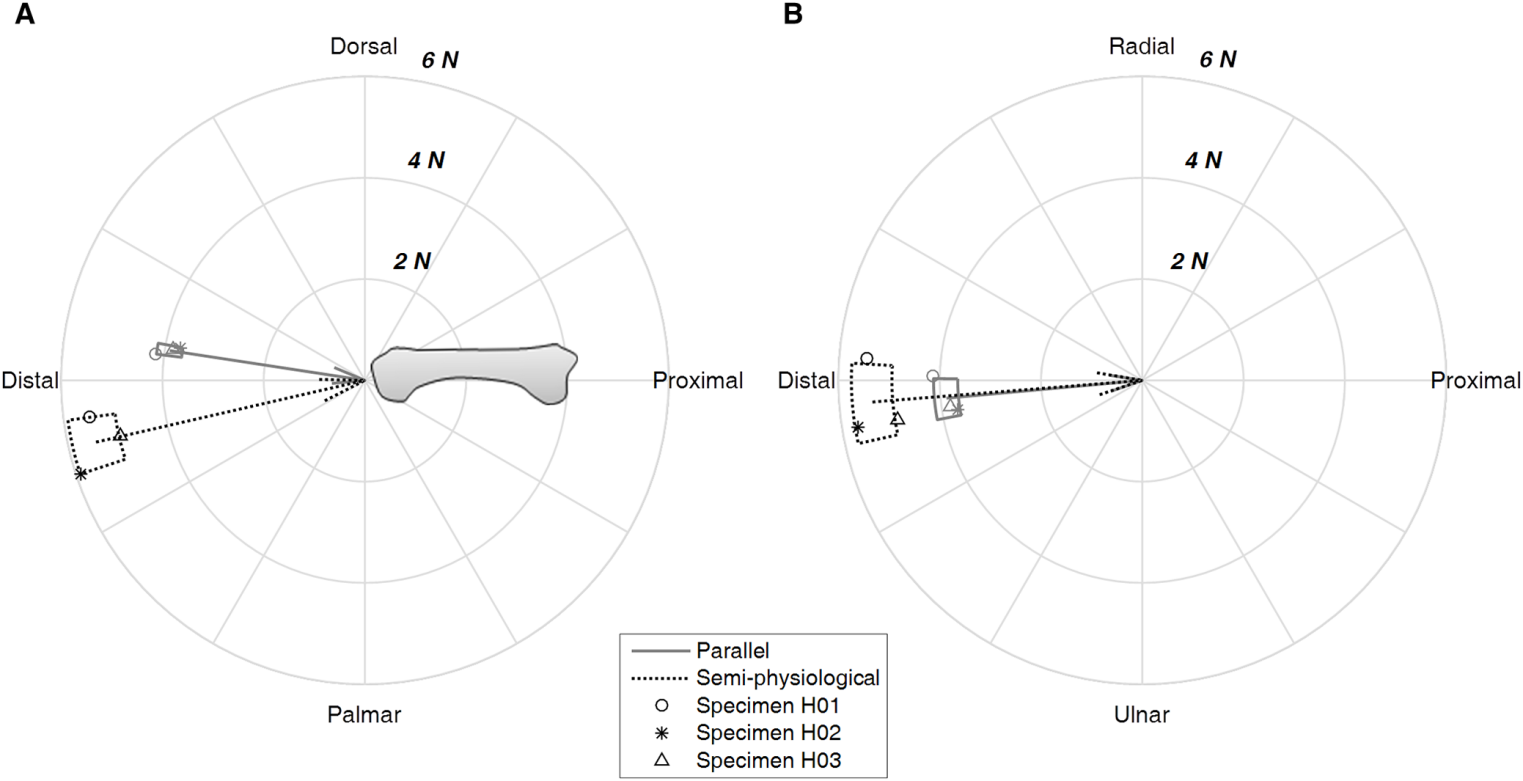
Net force acting on the metacarpal bone. The force is depicted in the flexion-extension plane (A) and the radial-ulnar deviation plane (B) with respect to the third metacarpal bone. The arrow shows the mean value of the three specimens and the box shows the range of one standard deviation. Data of each specimen are also presented.

The net force acting on the third metacarpal increased when the tendon path was changed from parallel to the semi-physiological condition. The resultant force increased from 3.93 ± 0.23 N in the parallel condition to 5.50 ± 0.47 N in the semi-physiological condition (Table 1). The force mainly loaded the metacarpal bone proximally in both tendon conditions, but the force was oriented palmarly in the parallel condition compared to dorsally in the semi-physiological condition (Fig. 4A). In the radial-ulnar direction, the tendon pathway did not alter the force direction. On average, the force was oriented radially. However, there was variation across the three specimens (Fig. 4B).

**Table 1:** Force experienced by the third metacarpal bone.

Tendon pathway	Proximal (+) distal (−)	Dorsal (+) palmar (−)	Radial (+) ulnar (−)	Resultant
Parallel	3.86 ± 0.26 N	−0.58 ± 0.07 N	0.34 ± 0.37 N	3.93 ± 0.23 N
Semi-physiological	5.29 ± 0.40 N	1.23 ± 0.57 N	0.42 ± 0.74 N	5.50 ± 0.47 N

**Note:**

The force experienced by the third metacarpal bone is presented in the dorsal-palmar, proximal-distal and radial-ulnar directions with respect to the metacarpal bone, and the resultant force magnitude is also presented. The force values are presented as mean ± standard deviation in Newtons.

### Strain of the metacarpal bone

**Figure 5:**
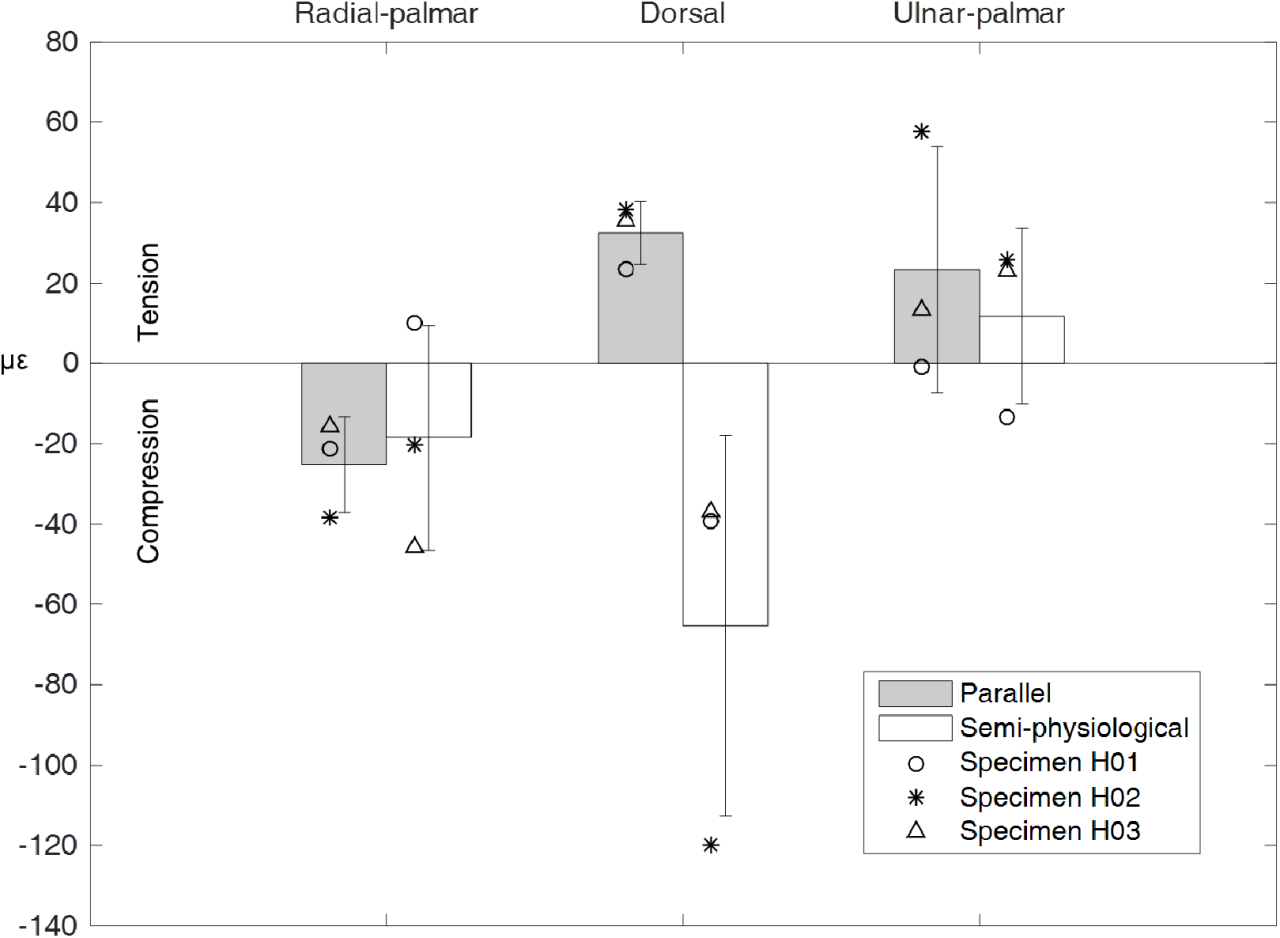
Metacarpal bone strain. The strain experienced by the three sides of the metacarpal bone is presented as the mean value (box) with one standard deviation (whiskers). Data of individual specimen are also presented. Positive value represents tension while negative value means compression.

The strain experienced by the dorsum of the metacarpal bone was consistent with the direction of the net force experienced by the metacarpal. In the parallel tendon condition, the dorsal side was in tension as the force was oriented palmarly, while in the semi-physiological condition, the dorsal side was in compression as the force was oriented dorsally (Figs. 4A and 5). The mean value of the tensile strain on the dorsal side across the three specimens was 32.50 ± 7.76 με, and the mean compressed strain was −65.36 ± 47.37 με. On average, the radial-palmar side was in compression while the ulnar-palmar side was in tension (Fig. 5). The strain measurement on the radial- and ulnar-palmar sides was consistent with the force measurement that, on average, the force experienced by the metacarpal bone was oriented radially (Fig. 4). However, variation in the strain pattern of the radial- and ulnar-palmar sides was observed across the three specimens.

### Fingertip force

**Figure 6:**
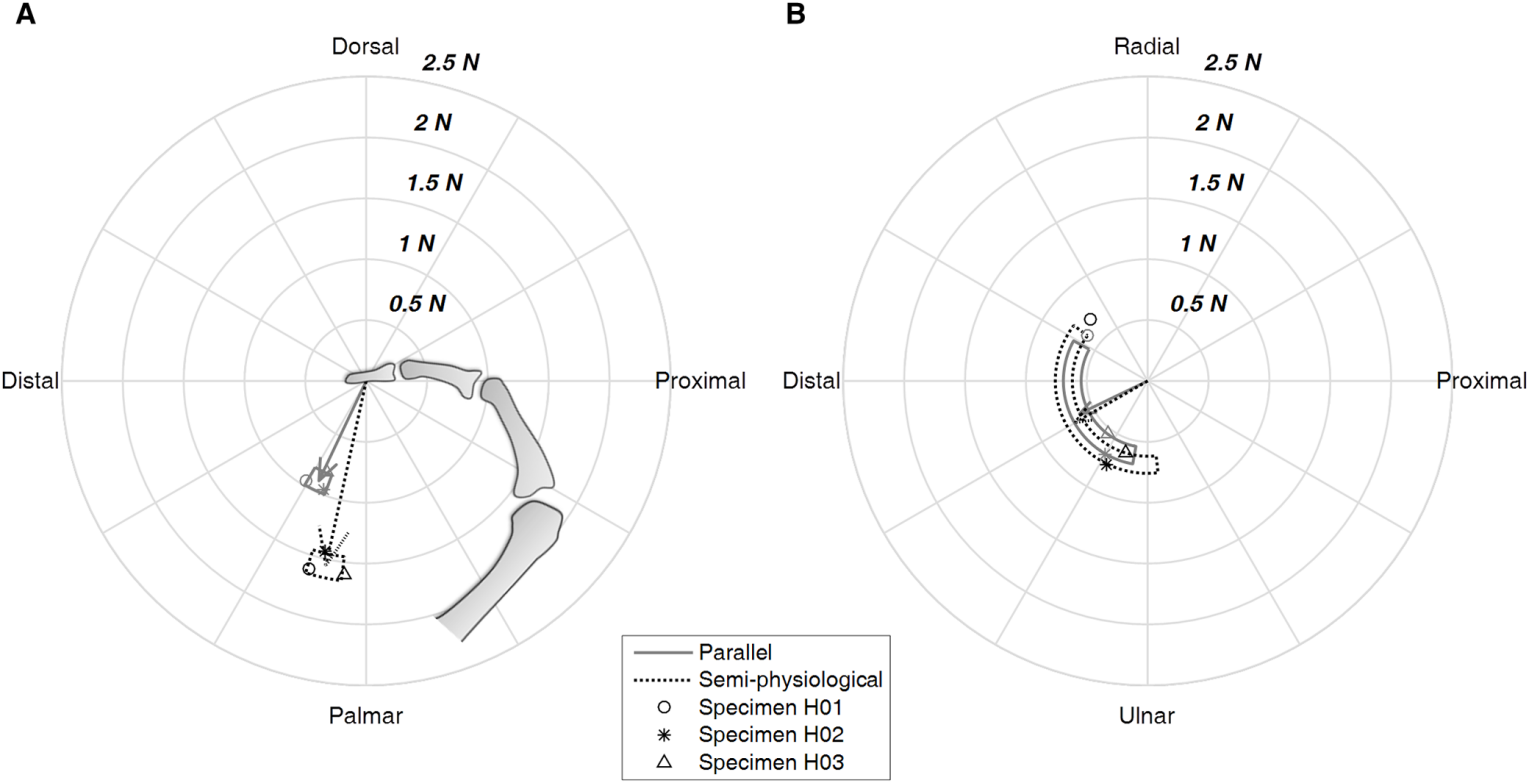
Fingertip force. The force is presented in the flexion-extension plane (A) and the radial-ulnar deviation plane (B) with respect to the distal phalanx. The arrow shows the mean value of the three specimens and the box shows the range of one standard deviation, and the data of individual specimen are also presented.

Similar to the pattern found for metacarpal force, fingertip force also increased as the tendon path changed. The mean value of the fingertip force across the three specimens increased from 1.02 ± 0.10 N in the parallel condition to 1.67 ± 0.06 N in the semi-physiological condition (Table 2), and the fingertip force mainly increased in the palmar direction. With respect to the orientation of distal phalanx, the fingertip force was oriented palmarly and distally in both conditions, but was more proximally-oriented in the semi-physiological condition (Fig. 6A). On average, the fingertip force was oriented ulnarly (Fig. 6B). However, similar to the metacarpal force results, there was variation across the three specimens (Fig. 6B).

**Table 2:** Fingertip force.

Tendon pathway	Proximal (+) distal (−)	Dorsal (+) palmar (−)	Radial (+) ulnar (−)	Resultant
Parallel	−0.39 ± 0.10 N	−0.82 ± 0.07 N	−0.22 ± 0.52 N	1.02 ± 0.10 N
Semi-physiological	−0.33 ± 0.14 N	−1.51 ± 0.10 N	−0.26 ± 0.66 N	1.67 ± 0.06 N

**Note:**

The fingertip force is presented in the dorsal-palmar, proximal-distal and radial-ulnar directions with respect to the distal phalanx. The resultant force magnitude is also presented, and the values are presented as mean ± standard deviation in Newtons.

## Discussion

We present a novel experimental design that is able to simultaneously measure in vitro force and strain experienced by the metacarpal bone without disrupting the MCP joint space. In addition, this experimental system is able to measure the fingertip force. This experimental approach builds upon previous in vitro studies have typically measured fingertip force only (Lee et al., 2008; Qiu & Kamper, 2014; Valero-Cuevas, Towles & Hentz, 2000). The results presented here are generally consistent with the, albeit limited, comparable data from previous studies and thus demonstrates the utility of this new experimental setup to provide biologically-relevant data to inform musculoskeletal and FE models of the hand.

### Effects of the tendon pathway modification

We found substantial variation the direction of net joint force and metacarpal shaft strain between the parallel and semi-physiological pathway conditions. Although force primarily loaded the metacarpal proximally in both tendon conditions, force was lower and oriented more palmarly in the parallel condition compared to higher force oriented dorsally in the semi-physiological condition. During the experiment, care was taken to avoid touching the metacarpal shaft when the metal bar was applied to change the tendon path from parallel to semi-physiological condition. However, we observed that the flexor tendons wrapped around the metacarpal head and pushed the bone dorsally, which may lead to the higher net force acting dorsally on the metacarpal bone in the semi-physiological condition. Accordingly, the strain experienced by the dorsum of the metacarpal changed from tension in the parallel condition to compression in the semi-physiological condition. In addition to the net force acting on the metacarpal bone, the fingertip force also increased in the semi-physiological condition, and the fingertip force was more proximally-oriented. The change in fingertip force magnitude and orientation may also be a result of the flexor tendons wrapping around the metacarpal head and applying additional force dorsally. The fingertip force increased even though the moment arm of the flexor tendons with respect to the MCP joint rotation centre decreased, suggesting a more efficient grasp function when the tendons run closer to the metacarpal bone shaft. This difference in force and strain between the two tendon pathway conditions also reveals the importance of tendon path simulation in computational models, which are typically modelled in a simplified manner similar to parallel condition (Kociolek & Keir, 2011; Lee et al., 2015).

### Variation across individuals

Variation in radial-ulnar force experienced by the metacarpal bone was observed in this study. There may be several reasons for this variation. Force in the radial-ulnar direction accounted for only 2–16% of the primary proximally-oriented force. The orientation of force in the radial-ulnar direction could be influenced by subtle variation in the asymmetry of the external shape of the metacarpal head (Drapeau, 2015; Susman, 1979) and/or the joint contact pattern at the MCP joint (Tamai et al., 1988). Analysis of the contact area at the human index MCP joint showed that the dorsal half of proximal phalanx base is in contact with the palmar side of the metacarpal head when the MCP is flexed to a similar degree (45°) as in this study (55°), but that the contact area is asymmetrical, being greater on the ulnar side of the joint (Tamai et al., 1988). Strain experienced by the metacarpal will also be affected by the internal structure of the bone, such as asymmetries in the cortical thickness of metacarpal shaft (Lazenby et al., 2011; Tsegai et al., 2017). Given the advanced age of all three anatomical specimens used in this study, it is highly likely that there may be substantial variation in bone density and cross-sectional geometry due to osteopenia (Hannan et al., 2000; Lips, Courpron & Meunier, 1978; Riggs, Khosla & Melton, 1998). Thus, age-related variability in bone structure and the high degree of asymmetry in human metacarpal and MCP joint morphology suggests that we might expect strong variation in the magnitude and direction of force and strain experienced by the human metacarpal bone. This inter-individual variation may have important implications for clinical treatment of joint disease or ergonomic studies, for example.

Our results on fingertip force are generally consistent with those of the few previous studies that have investigated in vitro fingertip force in a human finger (Qiu & Kamper, 2014; Valero-Cuevas, Towles & Hentz, 2000). These previous studies, however, focused on the human index finger, with intact soft tissue structures, and loaded one flexor tendon at a time at higher loads (10–30 N for FDP and 10–60 N for FDS in Valero-Cuevas, Towles & Hentz (2000); 8.2 N for FDP and 6.3 N for FDS in Qiu & Kamper (2014) vs. 2.9 N on each tendon in our study). Despite these differences, Qiu & Kamper (2014) also found that index fingertip force was oriented palmarly and distally when the finger was in a flexed posture (DIP 30°, PIP 60° and MCP 60°). Valero-Cuevas, Towles & Hentz (2000) positioned the index finger in a flexed posture (DIP 10°, PIP 45° and MCP 45°) and found that fingertip force was oriented palmarly and ulnarly, and variation was observed in the proximal-distal direction. The substantial variation in force orientation across the specimens observed in previous studies is comparable to the variation that we found, suggesting that individual variation in hand bone force transmission and the potential clinical implications require further investigation.

### Limitations and future considerations

This study has several limitations, with the small sample size and the in vitro testing condition as the most important factors. However, we consider three individuals to be sufficient to demonstrate the value of the newly developed experimental setup described here, which is able to simultaneously measure in vitro metacarpal force and strain. While the results of in vitro measurement might deviate from the in vivo condition, in vivo assessment of loading and deformation of hand bones is inaccessible with current technology and ethical regulations. In addition, this study not only quantified the force and strain experienced by the metacarpal bone but also measured the fingertip force, providing a more comprehensive data set for comparing the in vitro measurement of this study to the in vivo fingertip force within the literature (Quaine, Vigouroux & Martin, 2003; Shim et al., 2012; Vigouroux & Quaine, 2006). The force experienced by the metacarpal bone and the force at the fingertip were measured separately in this study because we only had access to one load cell. However, these two forces could be measured at the same time by implementing two load cells in the experimental setup.

Another potential limitation is the relatively low tendon loads (3% of the maximal muscle force) that were applied to the specimens to avoid rupture of the tendon, thus resulting in small force and strain measurements. Accuracy error of the sensors and the noise in the signals may bias the results. However, the accuracy error of the load cell was less than 0.05 N (see Supplemental Information). Also, the precision of strain gauge measurement was verified via beam models, and the analytical calculations showed the same strain pattern and similar values as the experimental results (see Supplemental Information).

This study used the same tendon load for three specimens while there might be individual difference in the anthropometry of the soft tissues. Due to the logistical challenges of measuring the PCSA of each specimen prior to the experiment, this study used the published mean PCSA values of the human FDS and FDP (Chao et al., 1989) to estimate the tendon load. The same tension was also applied to the FDS and FDP tendons, even though tension may vary between the two tendons during different activities (An et al., 1985). Previous studies have used controlled motors to apply tendon tension (Kutch & Valero-Cuevas, 2012; Shah et al., 2017), which could provide more biologically realistic simulations and could be considered in future studies. This study used weight to provide tendon tension, which is sufficient for the purpose of providing the comparable in vitro data for computational models.

Finally, the parallel and semi-physiological tendon pathways used in this study may not be ideal for simulating the tendon path within a hand with intact soft tissues. The parallel tendon pathway is a simplified condition that, although not fully representative of the biological condition, is particularly valuable for building informative musculoskeletal or FE models and obtaining replicable predictions. Although the artificial positioning of the tendons and the data extraction method could be improved, this study provides the best possible approximation of the normal physiological state within the current experimental constraints.

Although this study presents the force and strain measured in human third digit only, the experimental setup is adjustable to fit variation in the size and morphology of other human and nonhuman primate digits, as well as varying digit postures. Future studies could also include loading of more tendons and/or different loading patterns. Comparison among different digits with varied functional postures and tendon loading patterns may inform research on osteoarthritis, prosthetic design, and the interpretation of internal bone structure within the human hand. Comparative studies between humans and nonhuman primate digits can help inform the interpretation of the differences in external and internal bone morphology in relation to locomotor and manipulative behaviours. This comparative context is needed for more robust functional interpretations of fossil hominin hand morphology and the reconstruction of hand use throughout human evolution.

## Conclusions

This study presents a novel experimental design for simultaneous in vitro measurement of metacarpal force and strain. In addition, the experimental design allows for the quantification of fingertip force. Testing this experimental setup on three human cadaveric fingers revealed substantial difference in force and strain between two tendon pathway conditions, which, in turn, emphasizes the importance of tendon path simulation in computational models. The in vitro data presented in this study are not only useful for musculoskeletal and FE modelling in hand-related research such as prosthesis design, but also for the functional interpretation of the variation in internal bone structure within humans. In the future, investigation of forces and strain in different fingers could contribute to a better understanding of the varied prevalence of the MCP joint osteoarthritis across the digits (Caspi et al., 2001; Haugen et al., 2011). In the current study, the setup was applied to the human third finger in a single flexed posture, however, the setup can be used to experimentally test different human and nonhuman primate digits across a variety of a simulated grip postures. As such, this experimental setup allows for the quantification of force and strain during different manipulative and locomotor behaviour postures that are required to improve our understanding of primate hand function and adaptation.

## Supplemental Information

10.7717/peerj.5480/supp-1 Supplemental Information 1 Force and strain data. This table presents the force and strain data measured from the three specimens in two tendon path conditions, i.e. parallel and semi-physiological conditions. The force experienced by the third metacarpal bone (F_mc_) is presented in the proximal(+)/distal(−), dorsal(+)/volar(−), and radial(+)/ulnar(−) directions with respect to the metacarpal bone. The strain gauge (SG) measurement was performed at three sides of the bone, i.e. radial-palmar, dorsal, and ulnar-palmar sides. The fingertip force (F_tip_) is presented in the proximal(+)/distal(−), dorsal(+)/volar(−), and radial(+)/ulnar(−) directions with respect to the distal phalanx.

10.7717/peerj.5480/supp-2 Supplemental Information 2 Load cell accuracy. The load cell was tested before being used in the experiment. The error was less than 0.05 N and the full-scale accuracy error was less than 0.1%.

10.7717/peerj.5480/supp-3 Supplemental Information 3 Analytical and experimental strain values. The precision of strain gauge measurement was verified via beam models, and the analytical calculations showed the same strain pattern and similar values as the experimental results. Photo credit: Dr Szu-Ching Lu.

10.7717/peerj.5480/supp-4 Supplemental Information 4 MATLAB script for processing the force and strain data. The acquired force and strain signals were calibrated with baseline data collected when the flexor tendons were not loaded. Then, the signals were filtered using a sixth-order Butterworth low-pass filter with the cut-off frequency at 6 Hz.

## Additional Information and Declarations

### Data Availability

The following information was supplied regarding data availability: The raw data are provided in the Supplemental Files.
