# Supplementary material for: A novel experimental design for the measurement of metacarpal bone loading and deformation and fingertip force
Source: PeerJ. 2018 Sep 11;6:e5480. doi: 10.7717/peerj.5480 (PMC6138040; doi:10.7717/peerj.5480)
Supplement: Supplemental Information 2 — The load cell was tested before being used in the experiment. The error was less than 0.05 N and the full-scale accuracy error was less than 0.1%. [file peerj-06-5480-s002.pdf]

	Theoretical Value	Measured Value	Error	Full scale	Full-scale accuracy error
F_x	-1.87 N	-1.90 ± 0.03 N	0.03 ± 0.03 N	50 N	0.06 ± 0.06 %
F_y	0.87 N	0.89 ± 0.02 N	0.02 ± 0.02 N	50 N	0.04 ± 0.05 %
F_z	1.96 N	1.99 ± 0.01 N	0.03 ± 0.01 N	70 N	0.04 ± 0.01 %
