# Supplementary material for: A novel experimental design for the measurement of metacarpal bone loading and deformation and fingertip force
Source: PeerJ. 2018 Sep 11;6:e5480. doi: 10.7717/peerj.5480 (PMC6138040; doi:10.7717/peerj.5480)
Supplement: Supplemental Information 3 — The precision of strain gauge measurement was verified via beam models, and the analytical calculations showed the same strain pattern and similar values as the experimental results. Photo credit: Dr Szu-Ching Lu. [file peerj-06-5480-s003.pdf]

# STRAIN GAUGE MEASUREMENT

Three strain gauges (FLA-1-11-1L, Tokyo Sokki Kenkyujo Co., Ltd., Tokyo, Japan) were attached to the radial-palmar, dorsal, and ulnar-palmar sides of the third metacarpal bone at its mid-length.

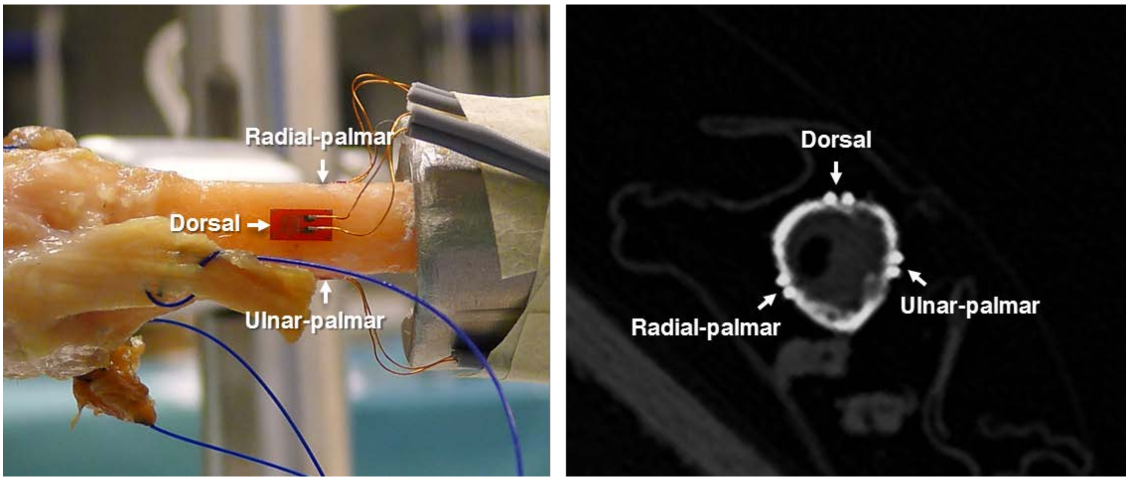


The acquired strain (and force/torque) values were averaged through a period of time. Here is an example of the synchronized data set of force, torque and strain measurement:

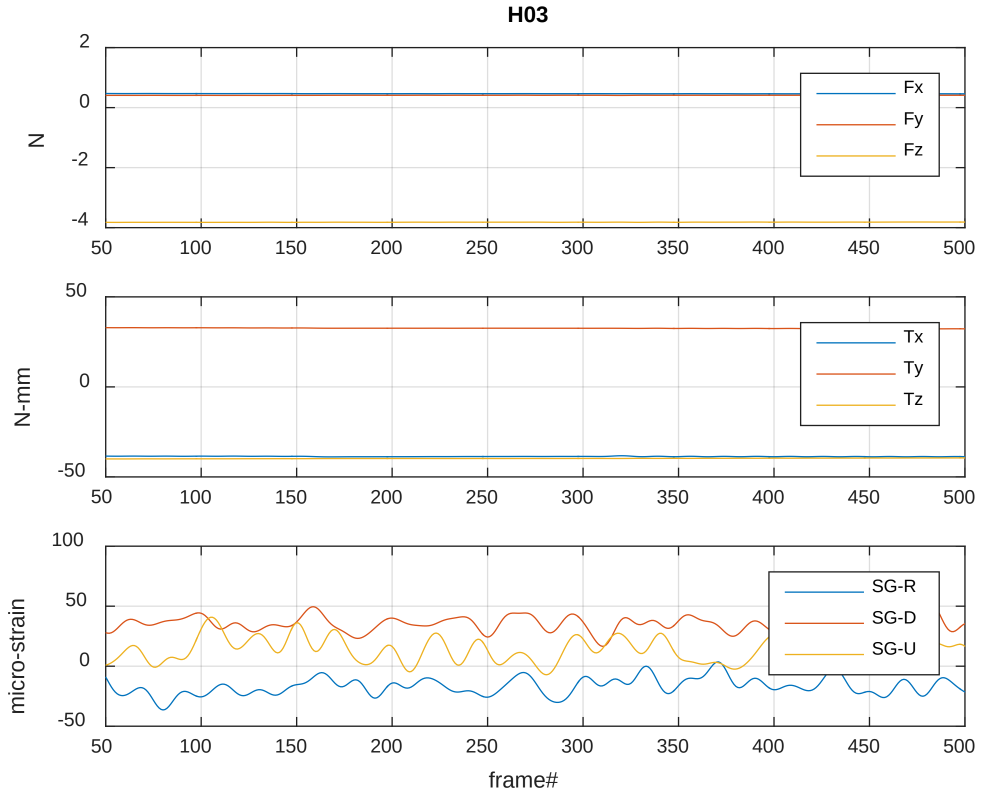

## ANALYTICAL MODEL

The load cell and strain gauges were digitized using MicroScribe (MicroScribe G2X Digitizer, Solution Technologies, Inc., MD, USA). These position data could be used to define the spatial relationship between the load cell and the cross-section of strain gauge measurement.

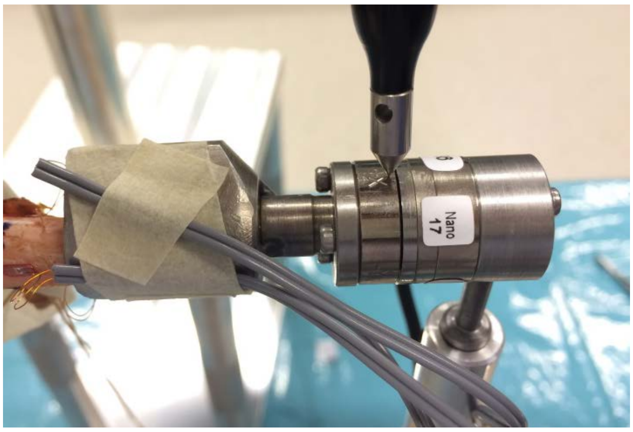


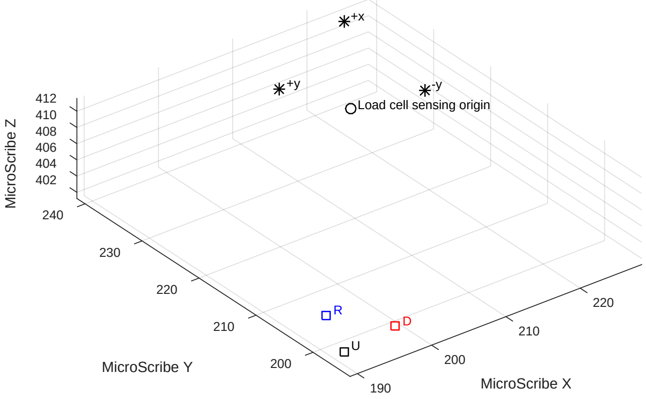


Medical CT images of the specimen (with strain gauges attached) were used to estimate the second moments of area. An annulus was used to approximate the cross-section of the bone.

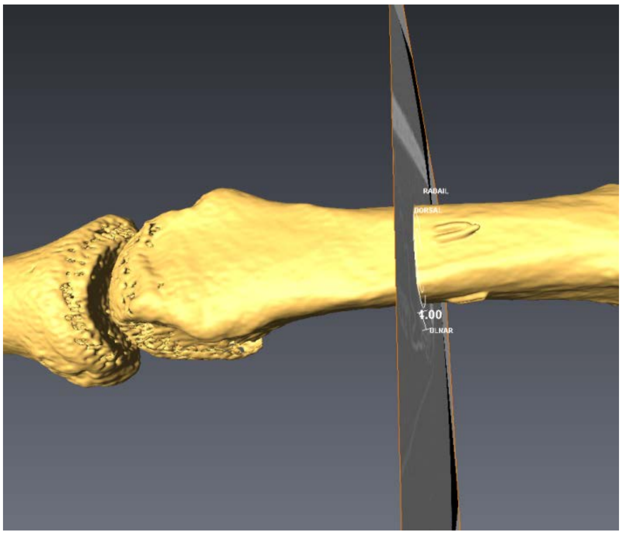

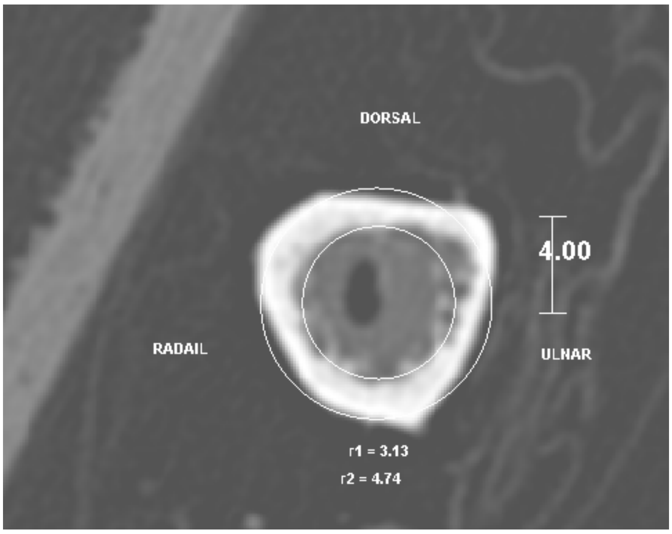


Equation for the analytical strain calculation:

$$\varepsilon(x,y) = \frac{1}{E} \times \left(-\frac{M_x}{I_{xx}} \times y + \frac{M_y}{I_{yy}} \times x - \frac{F_z}{A}\right)$$

The force and torque acting on the cross-section of interest were estimated using the acquired load cell and position data. The cross-section area and the second moments of area were estimated using CT images. A Young's Modulus of 18000 MPa (Nguyen et al., 2014) was adopted for calculation.

## ANALYTICAL STRAIN V.S. EXPERIMENTAL DATA

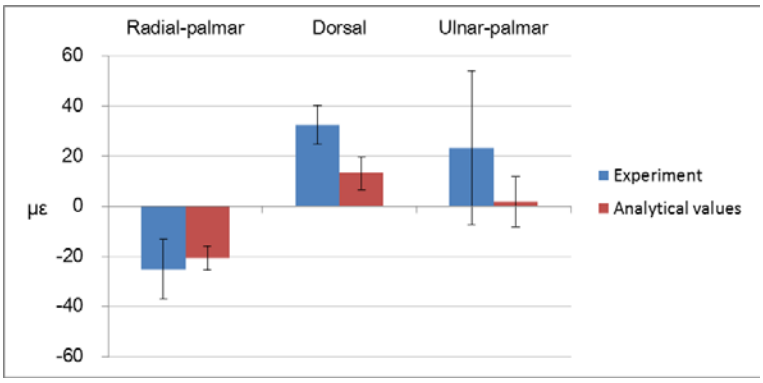


The difference between experimental data and analytical values could be due to several reasons. First, the positions of the load cell and strain gauges were digitized before the specimen was loaded. The bone position could be slightly changed after loading. Second, using an annulus to approximate the cross-section of the bone may also lead to biases in calculation of the area, second moments of area, and the center of the cross-section. Third, noise was observed in strain gauge signals. However, the experimental strain values were calculated as a mean over a period of time, which should decrease the effect of noise. Furthermore, the strain gauge measurement was also verified via cantilever beam test and duck bone experiment. Compared to human metacarpal bone, the cross-section of a duck bone was more similar to an annulus. Also, the loading condition in the duck bone experiment was more simplified than the human finger experiment. These could explain why the analytical values of the duck bone strain were more close to the experimental data than the human bone strain estimation.

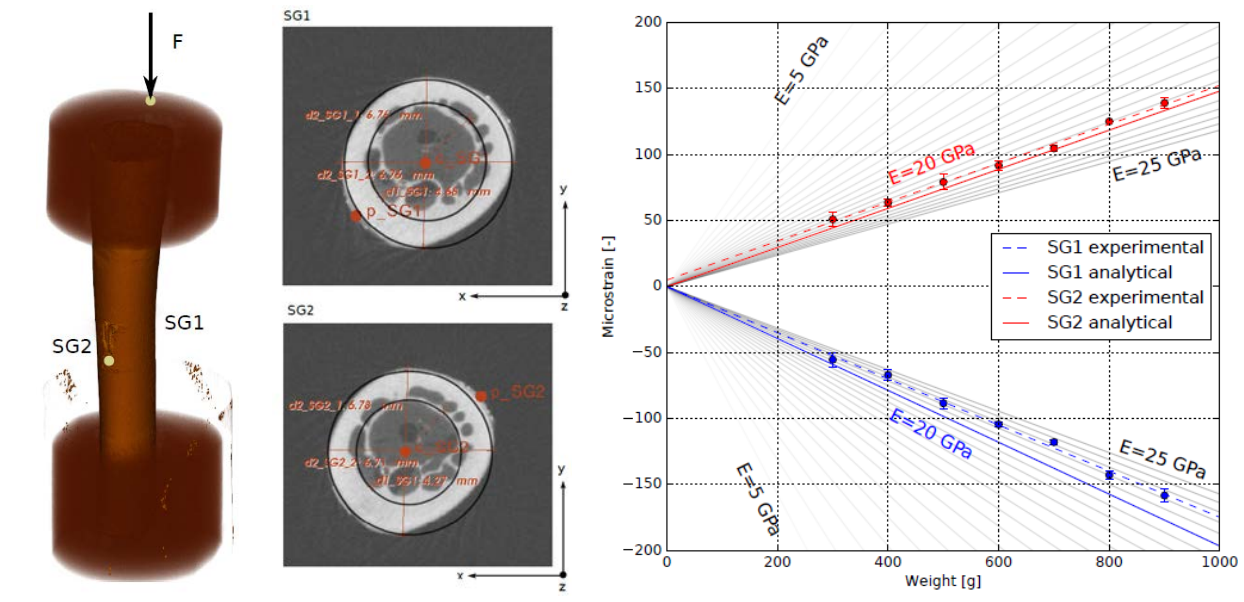


Duck bone experiment (by S.-C. Lu) and analytical estimation (by A. Synek)
