# Supplementary material for: A novel experimental design for the measurement of metacarpal bone loading and deformation and fingertip force
Source: PeerJ. 2018 Sep 11;6:e5480. doi: 10.7717/peerj.5480 (PMC6138040; doi:10.7717/peerj.5480)
Supplement: Supplemental Information 4 — The acquired force and strain signals were calibrated with baseline data collected when the flexor tendons were not loaded. Then, the signals were filtered using a sixth-order Butterworth low-pass filter with the cut-off frequency at 6 Hz. [file peerj-06-5480-s004.pdf]

```matlab
clc
clear all
close all

format long

%%% BASELINE TRIAL
B = A.Segment1.data;

%%% LOADED TRIAL
D = C.Segment1.data;

%% CALIBRATION MATRIX
CalMx = [0.0625204657   -0.0630916307   0.1288357306    -6.7033447424   -0.0837791216   6.5384584197
-0.4302057366   8.0100129269    0.0358277847    -3.9122009894   -0.0589201809   -3.7484428502
7.4946760221    0.2455069943    7.6705530794    0.0260664694    7.4017178908    -0.0668030005
-2.8060810974   49.0256828380   43.0001091913   -23.7084243012  -43.0948421200  -22.6657149189
-47.6415977028  -1.0411150414   24.8260560189   41.2221835138   24.4778848157   -40.3044497639
-2.0017325102   29.9349632526   -0.2162882720   28.4535507225   0.1426772357    28.7073211316 ];

%% FILTER
[a,b] = butter(6,6/50,'low'); % 6th order LPF with cutoff freq. at 6 Hz

%% DATA ACQUISITION RATE
f = 100;

%% LOAD CELL
LC0 = B(:,2:7); %%% BASELINE TRIAL
LC1 = D(:,2:7); %%% LOADED TRIAL

Bias = mean(LC0);

%%% CONVERT RAW DATA INTO FORCE AND TORQUES
LC2=[];
LC3=[];
for i=1:length(LC1);
   LC2(i,:) = LC1(i,:)-Bias;
   LC3(i,:) = (CalMx*LC2(i,:)')';
end
```

```
Fx = filtfilt(a,b,LC3(:,1));
Fy = filtfilt(a,b,LC3(:,2));
Fz = filtfilt(a,b,LC3(:,3));
Tx = filtfilt(a,b,LC3(:,4));
Ty = filtfilt(a,b,LC3(:,5));
Tz = filtfilt(a,b,LC3(:,6));

%%% DIRECTION
F_RU = Fx;
F_DV = Fy*(-1);
F_PD = Fz*(-1);

%% STRAIN GAUGES
SG0 = B(:,9:11); %%% BASELINE TRIAL
SG1 = D(:,9:11); %%% LOADED TRIAL

%%% REMOVE FIRST AND LAST 0.5-SEC DATA
%%% CALCULATE THE MEAN VALUE
offset = mean(SG0(51:length(SG0)-50,:));

%%% OFFSET
%%% MICRO-STRAIN
R1 = (SG1(:,1)-offset(1))*1000000;
D1 = (SG1(:,2)-offset(2))*1000000;
U1 = (SG1(:,3)-offset(3))*1000000;

%%% FILTER
SG_R = filtfilt(a,b,R1);
SG_D = filtfilt(a,b,D1);
SG_U = filtfilt(a,b,U1);
```
